# Supplementary material for: The French press: a repeatable and high-throughput approach to exercising zebrafish (Danio rerio)
Source: PeerJ. 2018 Jan 17;6:e4292. doi: 10.7717/peerj.4292 (PMC5775754; doi:10.7717/peerj.4292)
Supplement: Supplemental Information 1 — French press exercise protocol and tables [file peerj-06-4292-s001.docx]

**Supplementary Information**

1. French press exercise protocol

2. Tables (Table S1-S2)

3. Figures (Figure S1-S4)

**1. French press exercise protocol**

*Materials required:*

1. Coffee plunger (volume = 0.4 ℓ; diameter = 8 cm; Fig. S1 A)
2. Magnetic stir bar (45 x 7 mm; Fig. S1 B)
3. Single-plate magnetic stirrer (Fig. S1 C)
4. Multi-plate magnetic stirrer (Fig. S1 D and E)
5. Temperature probe
6. Stopwatch or metronome

*Set-up:*

1. Measure temperature of system water to be used for the trial. Water should be replaced as necessary between trials to keep water temperature constant within trials.
2. Place a magnetic stir bar at the bottom of each coffee plunger. Fill coffee plunger around 75% full with system water (Fig. S2 A). Switch on the magnetic stirrer and place the coffee plunger on top. Make sure that the stir bar is aligned in the centre of the coffee plunger (Fig. S2 B).
3. Lift the plunger slightly and place zebrafish into the coffee plunger using a fish net and funnel (Fig. S2 C). The zebrafish will swim above the plunger mesh* (Fig. S2 D).
4. Close lid fully and push the plunger down around half-way, leaving enough room for zebrafish to swim. Plunger height should be standardized within and between trials by propping up the plunger using a vertical pipette tip.
5. To remove zebrafish, pull the plunger all the way up to trap zebrafish between the lid and the mesh. Keeping the lid down, lift the plunger out of the coffee pot and release zebrafish through the spout into its holding container.

*Coffee plungers may come with two layers of mesh. For our experiment, we removed the fine-grade mesh prior to set-up. Removing the finer grade mesh increased the water current speed experienced by the zebrafish, which was required for assessing maximal swimming performance.

*Maximal swimming performance:*

1. Set a single-plate magnetic stirrer at a low speed (e.g. 200 rpm; Fig. S2 E). Although zebrafish may not swim against the current at very low speeds, this allows time for the zebrafish to habituate to the exercise system.
2. Using a stopwatch or metronome, increase the speed at 10 rpm intervals every 5 seconds. Make sure to carefully observe zebrafish swimming at all times. As you approach maximum swimming speed, zebrafish will begin to be swept into the water vortex. However, zebrafish may only lose control temporarily and may recover and continue to swim. If recovery occurs within 5 seconds, then continue to increase the speed in 10 rpm intervals.
3. At maximum swimming speed, the zebrafish spins out of control for 5 seconds. Stop the magnetic stirrer at this point and take note of this speed. Repeat for all zebrafish**.

**It is possible to set up two single-plate magnetic stirrers for each observer to simultaneously measure maximum swimming speeds of two zebrafish. With this set up, we estimate that 240 zebrafish can be measured in 5 hours with three observers (i.e. each measurement will last on average 7.5 minutes, including entry and removal of zebrafish).

*Exercise training:*

1. Using a multi-plate magnetic stirrer, determine a set exercise speed. Set exercise speed was intended to capture maximum sustained swimming ability and was determined during preliminary trials as the greatest speed at which all zebrafish could avoid being swept into the vortex.
2. Set up coffee plungers on top of the multi-plate magnetic stirrer and exercise zebrafish at set exercise speed for a set duration (e.g. 40 minutes; Fig. S2 F). When running the experiment, make sure to leave fish in a place of minimal disturbance (use covers if necessary).
3. Repeat this procedure as necessary for each zebrafish.

*General advice:*

1. Although zebrafish may not be motivated to swim at very low stirrer speeds, starting from low speeds allows time for zebrafish to habituate to the system and precludes erratic swimming behaviour at higher swimming speeds.
2. Water speed in the zebrafish swimming compartment will always be slower than the set stirrer speed. To obtain comparable estimates of swimming speeds across different French press units, water speeds as experienced by the zebrafish must always be calibrated before the experiment as described above (see Materials & Methods and Fig. S3).
3. During maximal swim performance trials, zebrafish swimming must always be carefully monitored. Often, zebrafish may temporarily be swept up into the water vortex at sub-maximal speeds and will recover from the vortex within 5 seconds. It is thus convenient to use a metronome to keep time and allow stirrer speed to be increased in a systematic manner.
4. Some zebrafish may rest on the bottom of the plunger during maximal swim performance trials and thus the frequency and duration of resting time must be accounted for.


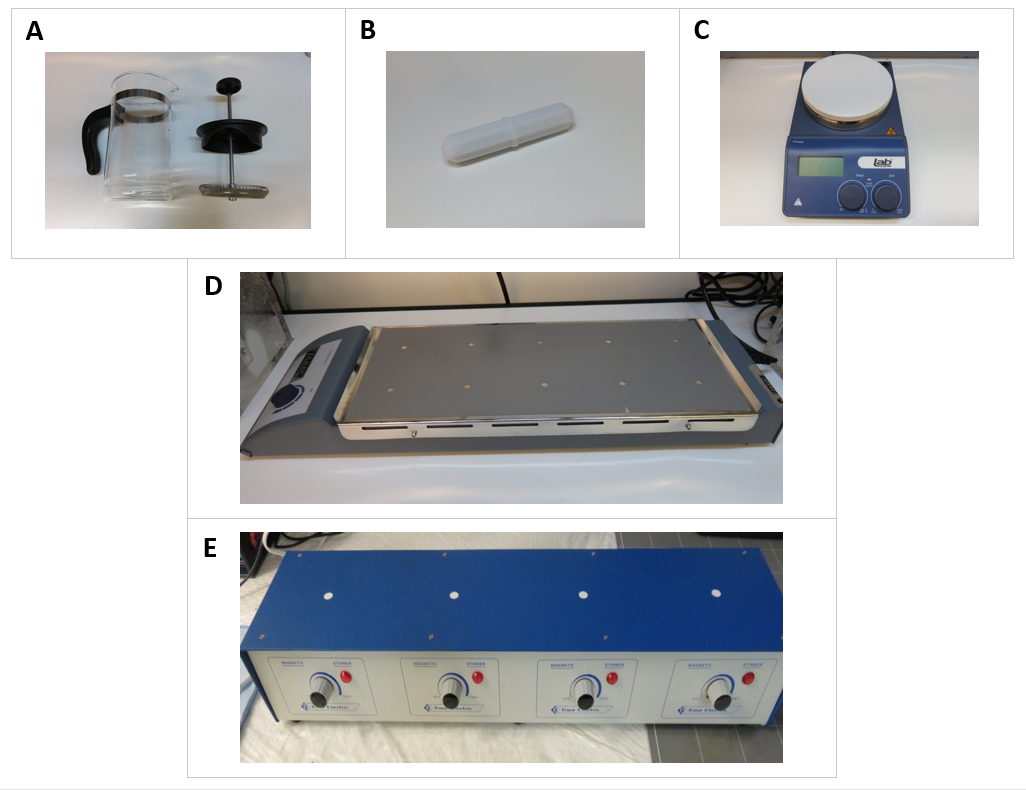


**Figure S1 Materials required for French press exercise.** (A) IKEA coffee plunger (B) Magnetic stir bar (C) LABTEK single-plate magnetic stirrer (D) LABTEK 10-plate magnetic stirrer (E) FRIED ELECTRIC 4-plate magnetic stirrer.


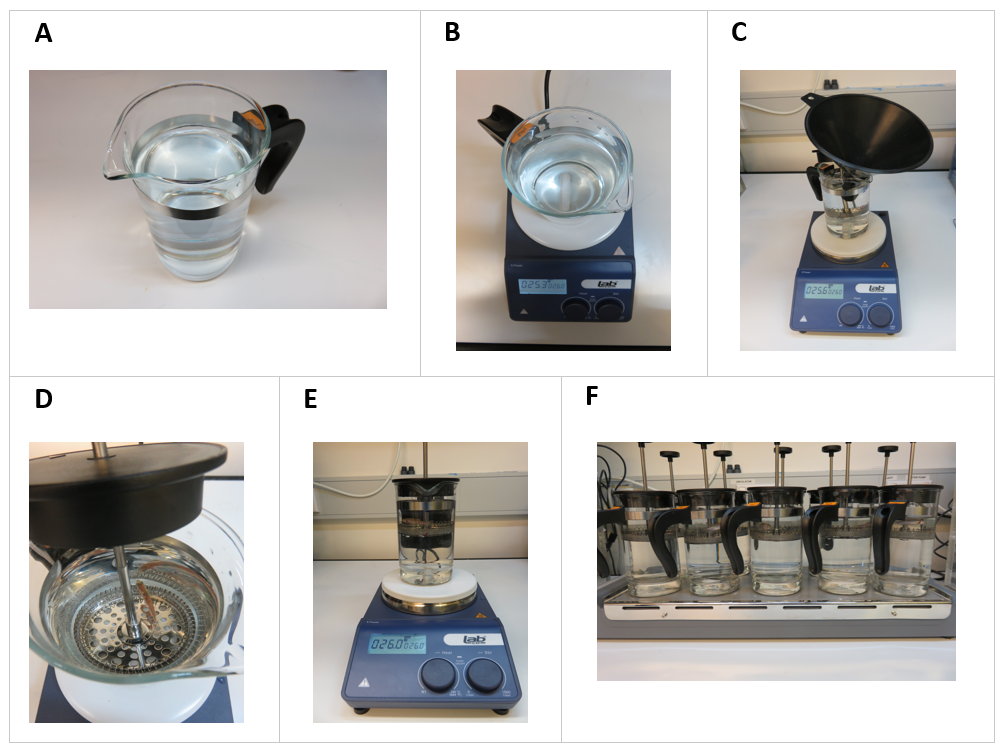


**Figure S2 Set-up of French press exercise.** (A) Coffee plunger with a magnetic stir bar was 75% filled with water (B) Coffee plunger set on top of a single-plate magnetic stirrer (C) Funnel positioned for entry of zebrafish to coffee plunger (D) Zebrafish swimming in the coffee plunger above the mesh (E) French press system set up for maximal swimming performance on a single plate magnetic stirrer (F) French press system set up for exercise training on a 10-plate magnetic stirrer.

**2. Tables**

**Table S1 Classification and coding of variables used in mixed-effects models.**

| Variable | Data type | Units/Levels |
| --- | --- | --- |
| Maximum swimming speed | Continuous | rpm |
| Mass | Continuous | g |
| Sex | Categorical | Male; Female |
| Length | Continuous | cm |
| Treatment | Categorical | Experimental; Control |
| Measure | Categorical | First; Second |
| Water temperature | Continuous | °C |

**Table S2 Model coefficients from linear mixed-effects model investigating change in zebrafish weight (g) after routine, sustained exercise.** Fixed effect intercept represents (i) first measure, (ii) experimental group, and (iii) female sex. Slope estimates, 95% lower (LCI) and upper (UCI) confidence intervals, *t*-values (*t*), degrees of freedom (*df*) and P-values (*P*) are reported.

| Fixed effects | Estimate | LCI | UCI | *t* | *df* | *P* |
| --- | --- | --- | --- | --- | --- | --- |
| Intercept | 0.616 | 0.569 | 0.662 | 25.312 | 38.00 | <0.001 |
| Measure (second) | –0.012 | –0.023 | –0.001 | –2.150 | 38.00 | 0.038 |
| Treatment (control) | –0.039 | –0.098 | 0.020 | –1.285 | 38.29 | 0.206 |
| Sex (male) | –0.110 | –0.168 | –0.051 | –3.628 | 37.00 | <0.001 |
| Measure by treatment (second*control) | 0.010 | –0.006 | 0.025 | 1.219 | 38.00 | 0.230 |

| Random effects | Variance (σ^2^) | SD (σ) | LCI (σ) |  | UCI (σ) |
| --- | --- | --- | --- | --- | --- |
| Individual fish ID | 0.008 | 0.093 | 0.072 |  | 0.113 |
| Residual | 0.000 | 0.018 | 0.014 |  | 0.022 |


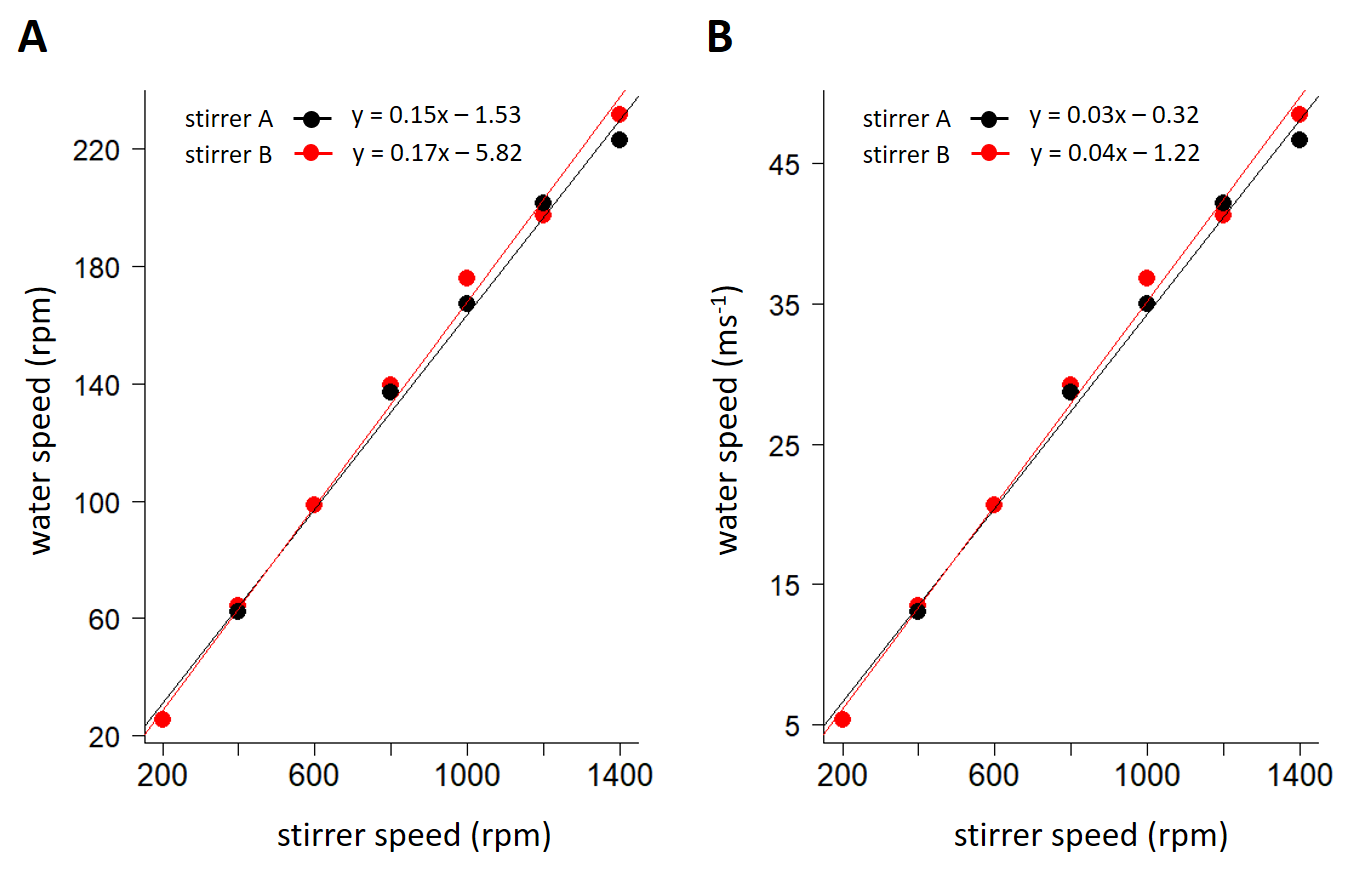
**3. Figures**

**Figure S3 Relationship between water speed and stirrer speed for two French press units.** (A) Water speed estimates (circles; rpm) in the zebrafish swimming compartment were obtained for stirrer speeds from 200 to 1200 rpm for two French press units. (B) Water speed estimates given in ms^-1^, as estimated for the midpoint of the radius of the coffee plunger. Plotted lines represent linear regressions.


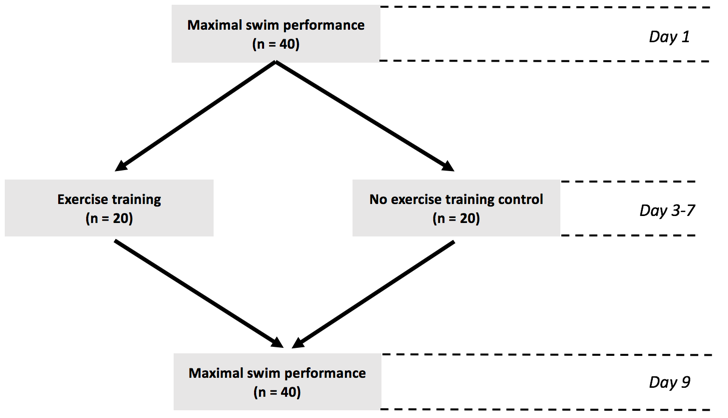


**Figure S4 Experimental timeline.** Initial maximal swim performance trials were conducted on 40 zebrafish after which fish were split into treatment (exercise trained) and control (untrained) groups. Exercise training regime followed for 5 days in the treatment group. Finally, maximal swim performance trials were conducted again for all fish to assess repeatability and differences in swim speed.
